# Effect of cellular and extracellular pathology assessed by T1 mapping on regional contractile function in hypertrophic cardiomyopathy

**DOI:** 10.1186/s12968-017-0334-x

**Published:** 2017-02-20

**Authors:** Peter P. Swoboda, Adam K. McDiarmid, Bara Erhayiem, Graham R. Law, Pankaj Garg, David A. Broadbent, David P. Ripley, Tarique A. Musa, Laura E. Dobson, James R. Foley, Graham J. Fent, Stephen P. Page, John P. Greenwood, Sven Plein

**Affiliations:** 1 Multidisciplinary Cardiovascular Research Centre (MCRC) & Leeds Institute of Cardiovascular and Metabolic Medicine, University of Leeds, Clarendon Way, Leeds, LS2 9JT UK; 2 Division of Epidemiology and Biostatistics, Leeds Institute of Cardiovascular and Metabolic Medicine, University of Leeds, Leeds, UK; 3 Department of Medical Physics and Engineering, Leeds Teaching Hospitals NHS Trust, Leeds, UK; 4 Inherited Cardiovascular Conditions Service, Leeds General Infirmary, Leeds, LS1 3EX UK

**Keywords:** Hypertrophic cardiomyopathy, T1 mapping, Extracellular volume, Strain, Tissue tagging, Feature tracking

## Abstract

**Background:**

Regional contractile dysfunction is a frequent finding in hypertrophic cardiomyopathy (HCM). We aimed to investigate the contribution of different tissue characteristics in HCM to regional contractile dysfunction.

**Methods:**

We prospectively recruited 50 patients with HCM who underwent cardiovascular magnetic resonance (CMR) studies at 3.0 T including cine imaging, T1 mapping and late gadolinium enhancement (LGE) imaging. For each segment of the American Heart Association model segment thickness, native T1, extracellular volume (ECV), presence of LGE and regional strain (by feature tracking and tissue tagging) were assessed. The relationship of segmental function, hypertrophy and tissue characteristics were determined using a mixed effects model, with random intercept for each patient.

**Results:**

Individually segment thickness, native T1, ECV and the presence of LGE all had significant associations with regional strain. The first multivariable model (segment thickness, LGE and ECV) demonstrated that all strain parameters were associated with segment thickness (*P* < 0.001 for all) but not ECV. LGE (Beta 2.603, *P* = 0.024) had a significant association with circumferential strain measured by tissue tagging.

In a second multivariable model (segment thickness, LGE and native T1) all strain parameters were associated with both segment thickness (*P* < 0.001 for all) and native T1 (*P* < 0.001 for all) but not LGE.

**Conclusion:**

Impairment of contractile function in HCM is predominantly associated with the degree of hypertrophy and native T1 but not markers of extracellular fibrosis (ECV or LGE). These findings suggest that impairment of contractility in HCM is mediated by mechanisms other than extracellular expansion that include cellular changes in structure and function. The cellular mechanisms leading to increased native T1 and its prognostic significance remain to be established.

## Background

Hypertrophic cardiomyopathy (HCM) is commonly defined as a disease of hypertrophy of the left ventricle (LV) in the absence of another cardiac or systemic cause [1]. Various patterns of hypertrophy are recognised [2] including widespread, asymmetric or eccentric myocardial thickening although most patients have a significant proportion of myocardium that is spared from overt hypertrophy [3].

HCM is most commonly caused by mutation in genes encoding proteins within the unit of myocardial contraction, the sarcomere [4]. The mechanisms that lead from dysfunction of the sarcomere to overt hypertrophy are complex and not as yet fully understood. Potential mechanisms include impaired calcium cycling, interstitial fibrosis, disturbed biomechanical stress sensing and microvascular dysfunction [5].

Previous studies predominantly performed with echocardiography speckle tracking have shown that there is widespread variation in myocardial contractility throughout the ventricle in HCM. Regional impairment of contractility is predominantly related to the extent of hypertrophy and presence of replacement fibrosis [6, 7]. Current guidelines therefore recommend that strain imaging could be used to investigate unexplained left ventricular hypertrophy that is not diagnostic of HCM [1, 8]. Alternative methods to echocardiography for the assessment of regional strain are cardiovascular magnetic resonance (CMR) tagging [9, 10] and post processing feature tracking (FT) [11–13] which are both highly reproducible and show good agreement.

Using CMR it is also possible to assess the tissue characteristics of the HCM phenotype. After administration of gadolinium based contrast agent, which is exclusively extracellular, late gadolinium enhancement (LGE) imaging allows identification of areas of focal fibrosis. Histological validation has suggested that the presence of LGE in HCM reflects replacement fibrosis [14], which is progressive over the course of the disease [15] and is associated with an adverse prognosis [16, 17].

LGE is a qualitative technique relying upon contrast between tissue with and without fibrosis, and is therefore of limited use in the detection of diffuse fibrosis. For this purpose T1 mapping techniques are used that give a quantitative pixel-wise map of myocardial T1 values. This can be performed without contrast (native T1), or by measuring both pre and post contrast T1maps to compute pixel-wise map of extracellular volume fraction (ECV %) [18]. ECV has been validated in surgical samples in HCM and has been shown to correspond to the histologically measured extent of fibrosis [19, 20]. Native T1 is influenced by several factors including extracellular space expansion (as is ECV) but also intracellular iron, lipid and water content [21, 22]. Native T1 has also been shown to be elevated in HCM [23] and both native T1 and ECV are elevated in genotype positive patients without overt hypertrophy [24, 25].

It is presently unknown which tissue characteristics are associated with contractile dysfunction in HCM. We therefore planned to assess native T1, ECV and LGE segmentally and quantify their association with strain measured in the same segment. This would provide insight into the pathological processes that lead to impaired contractility in HCM.

## Methods

### Enrolment criteria

Fifty consecutive patients with HCM were prospectively recruited from the local Inherited Cardiovascular Conditions Service between August 2014 and August 2015 and healthy controls (*N* = 30) were also recruited. The diagnosis of HCM was made independently by clinicians in keeping with current guidelines and based upon imaging including CMR, ECG, exercise testing, family history and genetic testing if possible [1, 8]. Exclusion criteria were previous surgical myomectomy, previous septal ablation, atrial fibrillation, previous myocardial infarction, uncontrolled hypertension, permanent pacemaker, defibrillator or other contraindication to CMR. Healthy controls had no existing medical conditions and were not taking any regular medication were also recruited to establish the normal range of ECV using the identical CMR protocol.

### CMR protocol

All subjects underwent an identical CMR protocol performed on a 3.0 Tesla Philips Achieva TX system (Philips, Best, The Netherlands) equipped with a 32 channel cardiac phased array receiver coil. A full blood count, including haematocrit was measured at the time of intravenous cannulation. The cardiac long and short axes were determined using standard scout views. Basal, mid and apical pre-contrast (native) short axis T1 maps were generated using a validated Modified Look Locker Inversion (MOLLI) protocol [26] (ECG triggered 5b (3 s) 3b MOLLI scheme with voxel size of 1.98 x 1.98 mm^2^, slice thickness 10 mm) and were planned using the 3 of 5 method [27]. Tissue tagging using a spatial modulation of magnetization (SPAMM) pulse sequence (spatial resolution 1.51x1.57x10mm^3^, tag separation 7 mm, ≥18 phases, typical TR/TE 5.8/3.5 ms, flip angle 10°, typical temporal resolution 55 ms) was acquired in the same three short axis slices in 34/50 patients. Left ventricular volumes were obtained from cine imaging covering the entire LV in the short axis: balanced steady state free precession (SSFP), voxel size 1.2 x 1.2 mm^2^, slice thickness 10 mm with no gap, 50 cardiac phases. Left atrial (LA) volumes were obtained from cine imaging covering the entire heart in the transverse axis: balanced SSFP, voxel size 1.2 x 1.2 mm^2^, slice thickness 6 mm with no gap, 50 cardiac phases. 0.15 mmol/Kg Gadovist (Bayer Schering) was delivered by power injector (Medrad Inc, Warrendale, Pennsylvania, USA) as a single bolus via a cannula placed in the ante-cubital fossa followed by 20 ml saline flush. Typical parameters for LGE were TR/TE 3.5/2.0 ms, flip angle 25°, acquired spatial resolution 1.54x1.76x10mm^3^ and performed in 10-12 short axis slices with ≥3 long axis orientations and phase-swapped acquisitions if indicated. Post contrast T1 mapping, using the same 5b (3 s) 3b MOLLI scheme as for native T1 mapping, was carried out in the same three slices exactly 15 min following last contrast injection (as above).

### CMR interpretation

Analysis was carried out using CVI42 (Circle Cardiovascular Imaging Inc. Calgary, Canada) and inTag (v1.0, CREATIS lab, Lyon, France) by two physicians blinded to clinical data. LV mass, end diastolic volumes (EDV), end systolic volume (ESV) and LV ejection fraction (EF) were measured from short axis cine images excluding papillary muscles and trabeculations. Native and post contrast T1 relaxation time of myocardium and blood pool were measured from the three scanner generated T1 maps by contouring a region of interest in each segment of the 16 segment American Heart Association (AHA) model [28]. ECV was calculated from native and post contrast T1 times of myocardium and blood pool and haematocrit as previously reported [19]. Segment thickness was measured for each AHA segment from the identical end-diastolic SSFP cine images corresponding to the T1 maps. Feature tracking analysis was carried out on the same images by drawing endocardial and epicardial contours and circumferential (Ecc-FT) and radial (Err-FT) strain calculated for each AHA segment. The reproducibility of Ecc-FT and Err-FT tested in 30 segments from 6 patients was good (intraobserver Ecc-FT 11.0%, Err-FT 7.7% and interobserver Ecc-FT 13.4%, Err-FT 10.1%).

For tagging analysis endocardial and epicardial contours were drawn on the short axis SPAMM sequences. Peak circumferential strain was measured for each segment of the AHA model using inTag (v1.0, CREATIS lab, Lyon, France). Strain was measured in the mid-myocardial layer (by disregarding epicardial and endocardial layers) which has previously been reported to be the most reproducible [10].

Segments were defined as hypertrophied if the maximal thickness was ≥15 mm in keeping with current guidelines [1, 8]. Replacement fibrosis was defined on a binary scale as the presence of LGE reported by 2 physicians experienced in CMR interpretation for each segment. All analyses were carried out blinded to the results of strain data.

### Statistical analysis

Statistical analysis was performed using Stata 14 (StataCorp, 2015, College Station, TX). Continuous variables are expressed as means ± standard deviation (SD). Categorical variables are expressed as N (%). Shapiro-Wilk test was used to test normality then unpaired *T* test or Mann Whitney *U* test used as appropriate. A mixed effects model with random intercept for each person was used to assess the association between Ecc-FT, Err-FT and Ecc-SPAMM and segment thickness, native T1, ECV and LGE. For each patient 16 segments with each parameter were included in the model to include segments with varying degrees of phenotypic expression. This model was used to account for the fact that segments from the same patient may not behave independently. The association between each strain parameter and segment thickness, native T1, ECV and LGE was tested using a two multivariable mixed effects model with random intercept adjusting for all three independent variables. ECV is not statistically independent from native T1 and therefore separate multivariable models were tested for each. Results are reported as mean ± standard deviation. *P* < 0.05 was considered statistically significant.

## Results

**Fig. 1:**
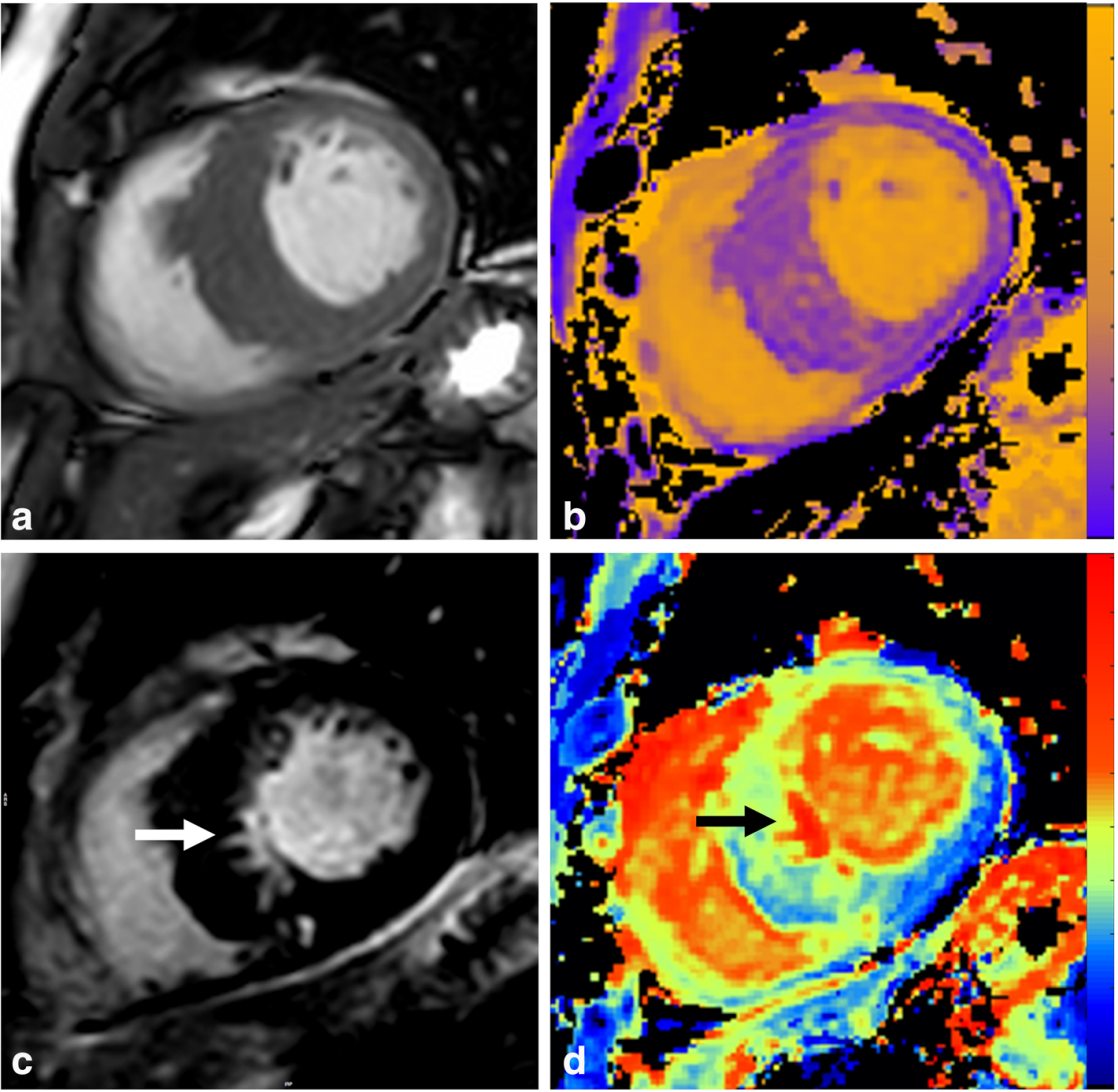
CMR images from a patient with asymmetric septal HCM. **a** SSFP imaging showing gross septal hypertrophy (>15 mm). **b** Native T1 map with colour scale ranging from 0 (purple) to 2000 ms (yellow). **c** Late gadolinium enhancement imaging showing a discrete area of replacement fibrosis in inferoseptum (white arrow). **d** ECV map ranging from 0 (blue) to 100% (red) confirming replacement fibrosis (black arrow)

Fifty patients with HCM were recruited. Patient characteristics are shown in Table 1. The distribution of hypertrophy was asymmetrical septal 36 (72%), concentric 5 (10%), mid cavity 4 (8%), apical 3 (6%) and isolated lateral 2 (4%). An example of cine imaging, native T1, LGE and ECV mapping from an identical slice of one patient is shown in Fig. 1. Segment thickness, LGE and tissue tagging could be analysed in all available segments. Native T1 could be analysed in 781/800 segments (median 1227 ms, interquartile range 1176-1271 ms) and ECV in 756/800 segments (median 27.4%, interquartile range 25.0-31.6%). Ecc-FT could not be analysed in 17/800 and Err-FT in 12/800 because of either artefact or poor tracking of myocardial features.

**Table 1:** Patient characteristics and findings

N	50	FH of HCM, n (%)	19 (38)
Male gender, n (%)	37 (74)	*CMR findings*	
Age, years	46.9 ± 11.7	LV EDV, ml/m^2^	78.7 ± 13.1
Height, cm	171.2 ± 9.0	LV EF, %	62.3 ± 5.9
Weight, kg	81.7 ± 14.6	LV Mass, g/m2	72.8 ± 26.3
Body mass index, kg/m^2^	27.7 ± 3.7	LAV, ml/m^2^	59.3 ± 13.5
Systolic blood pressure, mmHg	125.5 ± 17.6	LGE, n (%)	35 (70)
Diastolic blood pressure, mmHg	74.5 ± 12.5	*Medications*	
Heart rate	60.6 ± 10.2	Beta blocker, n (%)	24 (48)
*Echocardiography*		CC blocker, n (%)	8 (16)
Maximum wall thickness^a^ (mm)	19.3 ± 4.9	Disopyramide, n (%)	4 (8)
LVOT obstruction, n (%)	11 (22)	Diuretic, n (%)	2 (4)
Resting LVOT gradient, mmHg	68.5 ± 40.0		

*CC* calcium channel, *EDV* end diastolic volume indexed to body surface area, *EF* ejection fraction, *FH* family history, *LAV* left atrial volume indexed to body surface area, *LGE* late gadolinium enhancement, *LV* left ventricle, *LVOT* left ventricular outflow tract

^a^In 5 subjects with apical or localized lateral hypertrophy maximum wall thickness is taken from CMR

Controls were 36.2 ± 11.6 years old and 22 (73%) were male. From these controls the normal range for native T1 was 1190 ± 24.7 ms and ECV 24.3 ± 2.6%. Therefore segments with native T1 > 1239.4 ms and ECV > 29.5% (+2 SD) were defined as abnormally elevated.

The presence of hypertrophy, LGE, raised native T1 and raised ECV were all associated with impairment of all Ecc-FT, Err-FT and Ecc-SPAMM (*P* < 0.001 for all), Table 2.

**Table 2:** Strain according to segment thickness and tissue characteristics

	Segment thickness			LGE			Native T1			ECV		
	<15 mm	>15 mm	*P* value	-ve	+ve	*P* value	<1239.4 ms	>1239.4 ms	*P* value	<29.5%	>29.5%	*P* value
Ecc-FT (%)	−20.3 ± 8.3	−8.5 ± 8.6	<0.001	−20.1 ± 8.7	−10.6 ± 8.5	<0.001	−21.2 ± 8.1	−15.8 ± 9.7	<0.001	−20.0 ± 8.2	−17.0 ± 10.4	<0.001
Segments analysed	684	99		676	107		437	346		467	316	
Err-FT (%)	45.0 ± 27.2	15.2 ± 12.8	<0.001	44.8 ± 27.5	18.7 ± 15.5	<0.001	47.9 ± 27.4	32.7 ± 25.6	<0.001	43.3 ± 26.0	38.2 ± 29.7	<0.001
Segments analysed	689	99		681	107		441	347		468	320	
Ecc-SPAMM (%)	−20.5 ± 8.1	−11.1 ± 4.8	<0.001	−20.6 ± 8.0	−11.2 ± 4.7	<0.001	−21.6 ± 7.5	−16.9 ± 8.5	<0.001	−20.8 ± 7.8	−17.5 ± 8.7	<0.001
Segments analysed	494	48		488	54		320	222		349	193	

Strain measured by Ecc-FT, Err-FT and Ecc-SPAMM according to the presence of overt hypertrophy (>15 mm), late gadolinium enhancement (LGE), elevated native T1 (defined as 2SD above mean measured from healthy controls) and exracellular volume fraction (ECV, also defined as 2SD above mean measured from healthy controls)

### Mixed effects model with random intercept

On univariable analysis segment thickness, LGE, native T1 and ECV all had a significant association with Ecc-FT, Err-FT and Ecc-SPAMM, Table 3. In multivariable model 1 (segment thickness, LGE, ECV) only segment thickness had a significant association with Ecc-FT (Beta 1.317 (95% confidence interval (CI) 1.165;1.470), *P* < 0.001). In multivariable model 2 (segment thickness, LGE, native T1) both segment thickness (Beta 1.219 (95% CI 1.070;1.368) *P* < 0.001) and native T1 (Beta 0.015 (95% CI 0.008;0.022) *P* < 0.001) had significant association with Ecc-FT.

**Table 3:** Regression model to identify association between ﻿segmental strain, segement thickness and tissue characteristics

			Segment thickness	BIC	LGE	BIC	Native T1	BIC	Extracellular volume	BIC
Ecc-FT	Univariable	Beta	**1.325 (1.200;1.450)**	5345.6	**9.234** **(7.444;11.024)**	5592.9	**0.039 (0.031;0.047)**	5476.3	**0.268 (0.168;0.368)**	5368.1
		*P* value	**<0.001**		**<0.001**		**<0.001**		**<0.001**	
	Multivariable 1	Beta	**1.317 (1.165;1.470)**	5074.1	1.294 (-0.601;3.191)				−0.046 (-0.136;0.045)	
		*P* value	**<0.001**		0.195				0.394	
	Multivariable 2	Beta	**1.219 (1.070;1.368)**	5214.3	0.331 (-1.470;2.131)		**0.015 (0.008;0.022)**			
		*P* value	**<0.001**		0.718		**<0.001**			
Err-FT	Univariable	Beta	**−3.573 (−3.963;−3.183)**	7167.8	**−25.595 (-30.940;-20.251)**	7354.3	**−0.118 (−0.142;−0.093)**	7177.7	**−0.693 (−0.991;−0.394)**	6997.0
		*P* value	**<0.001**		**<0.001**		**<0.001**		**<0.001**	
	Multivariable 1	Beta	**−3.532 (-4.011;-3.073)**	6758.4	−4.861 (-10.661;0.941)				0.201 (-0.080;0.481)	
		*P* value	**<0.001**		0.101				0.161	
	Multivariable 2	Beta	**−3.193 (-3.659;-2.726)**	6986.5	−1.266 (-6.868;4.336)		**−0.053 (-0.075;-0.030)**			
		*P* value	**<0.001**		0.658		**<0.001**			
Ecc-SPAMM	Univariable	Beta	**1.039 (0.881;1.198)**	3651.4	**8.525 (6.382;10.667)**	3738.8	**0.033 (0.024;0.042)**	3681.3	**0.292 (0.167;0.417)**	3601.5
		*P* value	**<0.001**		**<0.001**		**<0.001**		**<0.001**	
	Multivariable 1	Beta	**0.964 (0.782;1.146)**	3451.0	**2.603 (0.341;4.866)**				0.061 (-0.055;0.177)	
		*P* value	**<0.001**		**0.024**				0.303	
	Multivariable 2	Beta	**0.0849 (0.669;1.030)**	3581.4	2.194 (-0.029;4.417)		**0.017 (0.008;0.025)**			
		*P* value	**<0.001**		0.053		**<0.001**			

Mixed effects model, with random intercept for each patient assessing the association between Ecc-FT, Err-FT and Ecc-SPAMM and the tissue characteristics of each segment. Multivariable model 1 includes segment thickness, LGE and ECV and model 2 includes segment thickness, LGE and native T1. Values denoted in bold are statistically significant with *P*<0.05. Beta, regression coefficient with 95% confidence interval; BIC, Bayesian information criterion

In multivariable model 1 only segment thickness had a significant association with Err-FT (Beta -3.532 (95% CI -4.011;-3.073), P < 0.001). In multivariable model 2 both segment thickness (Beta -3.193 (95% CI (-3.659;-2.726) *P* < 0.001) and native T1 (Beta -0.053 (95% CI -0.075;-0.030) *P* < 0.001) had significant association with Err-FT.

In multivariable model 1 both segment thickness (Beta 0.964 (95% CI 0.782;1.146), *P* < 0.001) and LGE (Beta 2.603 (95% CI 0.341;4.866), *P* = 0.024) had a significant association with Ecc-SPAMM. In multivariable model 2 both segment thickness (Beta 0.0849 (95% CI 0.669;1.030) *P* < 0.001) and native T1 (Beta 0.017 (95% CI 0.008;0.025) *P* < 0.001) but not LGE had significant association with Ecc-SPAMM.

### Influence of genotype

10/50 patients had presence of a genetic mutation known to cause HCM (MYBPC3 *N* = 7, MYH7 *N* = 4, TNNI3 *N* = 1). There was a trend to higher native T1 overtly hypertrophied segments (>15 mm, *N* = 99) in genotype positive vs genotype negative patients, 1295.9 ± 65.1 vs 1272.3 ± 57.9 ms, *P* = 0.055. In the same segments there was no significant difference in ECV in genotype positive vs genotype negative patients, 37.1 ± 10.1 vs 34.8 ± 10.2%, *P* = 0.23).

## Discussion

Hypertrophic cardiomyopathy is characterised by both cellular and extracellular pathological processes. With CMR it is possible to assess both gross macroscopic abnormalities, as reflected by segment thickness and replacement fibrosis on LGE, as well both the cellular and extracellular compartments at a microscopic level, by assessing ECV and native T1.

We have demonstrated that segmental contractility after correction for segment thickness has a significant association with myocardial native T1 but not LGE or ECV. ECV measurement assesses predominantly the extracellular compartment, whereas native T1 reflects the intracellular water, iron and lipid content [22]. These findings suggest that in HCM changes in the cellular structure and function rather than extracellular expansion may mediate impairment of myocardial contractility.

The cellular mechanisms that lead from mutation of sarcomeric proteins to increased native T1 and impaired contractile function remain to be established [5]. Native T1 could help elucidate these mechanisms by identifying the extent of cellular changes in patients with different disease causing mutations. It also has the potential to be a marker for monitoring disease progression, response to clinical intervention and long-term prognosis.

Ecc-SPAMM is the best validated CMR method for the assessment of strain [29, 30]. However it is hampered by tag fading in diastole and the relatively coarse tag separation of 7 mm may limit use in thinned regions of myocardium. We have therefore also assessed strain by FT which - although less well validated than tissue tagging - overcomes the issues of tag fading and its spatial resolution of 1.2x1.2 mm^2^ allows better assessment of thinned regions of myocardium than is possible with SPAMM. Both we and other groups have reported good reproducibility of strain measured by this method of FT. Reassuringly, both FT and SPAMM consistently demonstrated a relationship between native T1 in two orthogonal planes (circumferential and radial).

### T1 mapping

Cellular changes that may be detected in HCM include altered calcium cycling, impaired biomechanical stress sensing and disturbed cardiac energy homeostasis [31] and extracellular changes include myocyte disarray and fibrosis [32, 33]. The assessment of both native T1 and ECV by CMR offer the unique opportunity to assess changes in both the cellular and extracellular compartments non-invasively. ECV measured by T1 mapping has shown to be elevated in HCM and has a strong correlation with fibrosis measured histologically [19, 34].

There is already clear evidence that in HCM both native T1 [23, 35] and ECV [19, 36] are elevated. This increase can even be detected in those with sarcomeric mutations but without overt hypertrophy [24]. We have observed that for all three measures of regional strain native T1 but not ECV was associated with contractile function suggesting that changes in cellular rather than extracellular tissue characteristics contribute predominantly to loss of contractile function in HCM. An alternative explanation is that native T1 is a stronger marker of fibrosis than ECV. However this seems less likely given that native T1 reflects intracellular as well as extracellular signal and studies have demonstrated that native T1 has a much weaker association with histological measures of extracellular fibrosis than ECV [37, 38].

The major shortcoming of native T1 is that it varies significantly between field strengths, scanner vendor and technique used to measure it [39] and in clinical practice requires validation for the specific pulse sequence and field strength used [18]. We have previously reported excellent reproducibility in both phantom and human studies of the pulse sequence used in this study [40]. However native T1 has merits that would support its use in this clinical application. Unlike ECV its measurement does not require intravenous cannulation, administration of contrast, or a blood sample to measure haematocrit.

### Late gadolinium enhancement

We have reported, from multivariable model 2, that native T1 but not LGE has a significant association with impairment of regional function. LGE, like ECV, is directly influenced by the extent of extracellular fibrosis with a strong linear correlation between the extent of LGE within a particular segment and the amount of collagen measured histologically [14].

We identified that LGE, even after correction for segment thickness, had a significant association with Ecc-SPAMM (multivariable model 1) in keeping with previous studies which did not include T1 mapping [6, 7]. However, when native T1 was included (multivariable model 2) the association was no longer significant suggesting again that changes in cellular structure are more determinant in regional contractility.

In HCM, there is a strong correlation between extent of LGE and ECV expansion [36] although the association between native T1 and LGE is much weaker [25]. Increased native T1 can be detected in regions without LGE [35, 41] and is therefore a good discriminator between healthy and diseased myocardium [23]. The fact that native T1 influences contractile function but LGE does not adds to the existing literature in demonstrating that it is able to measure cellular changes that we have previously been unable to quantify.

### Segment thickness

We have also reported that segmental strain is strongly associated with segment thickness. These findings corroborate previous studies that have shown a relationship between degree of hypertrophy and presence of LGE and strain measured by speckle tracking echocardiography [6, 7] and feature tracking by CMR [42]. Dhillon et al. measured longitudinal strain by feature tracking echocardiography in subjects with HCM undergoing surgical myomectomy, also reporting histological findings and in vitro contractility of the surgically excised myocardium. They found that the degree of histological fibrosis correlated with both strain measured by echocardiography and in vitro [43]. However strain was measured in segments with severe hypertrophy causing LVOT obstruction and more than half of the specimens studied displayed small intramural coronary arteriole dysplasia which is known to correspond strongly with focal LGE detected by CMR [44]. Therefore the subjects studied were at the severe end of the hypertrophic spectrum and not directly comparable with subjects and segments in the present study with a wide range of phenotypic presentations.

### Limitations

The patients studied all had an established HCM with a diagnosis made according to current guidelines [1, 8]. Our findings cannot therefore be extrapolated to those without overt expression of HCM. The correlation of both native T1 and ECV with cellular and extracellular changes has not been tested histologically in the study however but has been done in previous studies [19, 34].

We have only measured strain from short axis measurements specifically because the strength of the segmental analysis that we have carried out is based on the fact that T1 maps, SSFP cines and LGE acquisitions can all be carried out in the identical short axis plane. Comparing strain from long axis cines to ECV from short axis maps would add an unacceptable degree of error.

## Conclusions

Regional strain impairment measured by feature tracking and SPAMM is predominantly associated with the degree of hypertrophy and native T1, but not extracellular fibrosis (LGE or ECV). These findings suggest that impairment of contractility in HCM may be mediated by mechanisms other than extracellular expansion that may include cellular changes in structure and function. The cellular mechanisms leading to increased native T1 and its prognostic significance remain to be established.

## Abbreviations

CMR: Cardiovascular magnetic resonance
ECV: Extracellular volume
EDV: End diastolic volume
EDVI: End diastolic volume indexed to BSA
EF: Ejection fraction
ESV: End systolic volume
FT: Feature tracking
HCM: Hypertrophic cardiomyopathy
LGE: Late gadolinium enhancement
LV: Left ventricle
LVMI: Left ventricle mass indexed to BSA
MOLLI: Modified Look-Locker inversion recovery
SPAMM: Spatial modulation of magnetization
SSFP: Steady-state free precession
T1: T1 tissue relaxation time
